# A Novel Sensor System for In Vivo Perception Reconstruction Based on Long Short-Term Memory Networks

**DOI:** 10.3390/s22197407

**Published:** 2022-09-29

**Authors:** Ding Han, Guozheng Yan, Lichao Wang, Fangfang Hua, Lin Yan

**Affiliations:** 1 School of Electronic Information and Electrical Engineering, Shanghai Jiao Tong University, Shanghai 200240, China; 2 Shanghai Engineering Research Center of Intelligent Addiction Treatment and Rehabilitation, Shanghai 200240, China; 3 Scientific Computing & Imaging Institute, University of Utah, Salt Lake City, UT 84112, USA

**Keywords:** strain gauge sensor, LSTM, perception reconstruction, artificial anal sphincter, fecal incontinence

## Abstract

Monitoring bodily pressure could provide valuable medical information for both doctors and patients. Long-term implantation of in vivo sensors is highly desirable in situations where perception reconstruction is needed. In particular, for fecal incontinence, artificial anal sphincters without perceptions could not remind patients when to defecate and even cause ischemic tissue necrosis due to uncontrolled clamping pressure. To address these issues, a novel self-packaging strain gauge sensor system is designed for in vivo perception reconstruction. In addition, long short-term memory (LSTM) networks, which show excellent performance in processing time series-related features and fitting properties, are used in this article to improve the prediction accuracy of the perception model. The proposed system has been tested and compared with the traditional linear regression (LR) approach using data from in vitro experiments. The results show that the Root-Mean-Square Error (RMSE) is reduced by more than 69%, which demonstrates that the prediction accuracy of the proposed LSTM model is higher than that of the LR model to reach a more accurate prediction of the amount of intestinal content. Furthermore, outcomes of in vivo experiments show that the robustness of the novel sensor system based on long short-term memory networks is verified through experiments with limited data.

## 1. Introduction

Fecal incontinence is a disorder of the defecation function of the anal sphincter, which seriously affects the daily life of patients [1]. Colostomy is a common treatment. However, the colostomy changes the original physiological structure of the intestine, causing the loss of the capability of the sphincter to control stool. Even worse, the self-esteem of patients is damaged, and social activities are also seriously affected [2,3].

Nowadays, artificial anal sphincters (AAS), as in vivo medical devices that could recover the function of sphincters, have been investigated by researchers all around the world [4,5]. The earliest artificial anal sphincter is inspired by artificial urinary sphincter, which is used to treat urinary incontinence [6]. At present, the main artificial anal sphincters in clinical use include the American artificial bowel sphincter (ABS) [7,8], the Austrian soft anal band system (SABS) [9], the British artificial puborectalis anal sphincter system (PAS) [10,11,12,13] and the French magnetic bead chain artificial anal sphincter (Magnetic Anal Sphincter, MAS) [14,15]. Some progress has been made by these researchers. These artificial anal sphincters have utilized clamping of the intestine to recover the function of maintaining continence. However, new problems have arisen. The artificial anal sphincters mentioned above could not recover the perception function of the sphincters. That is to say, they lack the ability to remind patients when to defecate. This usually causes tissue ischemic necrosis due to the lack of pressure feedback.

New explorations have been taken to solve these issues by researchers, though few studies can be found. Firstly, developing the habit of having multiple bowel movements per day has been proposed. This causes some relief to the tissue ischemic necrosis to some extent. However, increasing the number of bowel movements blindly goes against physiology and undoubtedly brings a heavy burden to patients. Therefore, perception reconstruction is considered necessary. In order to recover the perception function of anal sphincters, sensors have been studied by lots of groups as a solution. In reference [16], although the system’s accuracy is not high enough, the pressure of the intestinal canal and pressure of sensing cuff was positively correlated within a certain range. In addition, support vector machines (SVM) [17] and linear regression (LR) [18,19] are also applied to predict the pressure in the intestine. They tried to improve the prediction accuracy for the in vivo perception reconstruction system. However, the prediction of the amount of feces present in the intestine is challenging due to its nonlinear variation. The incorporation of the intestinal surroundings makes it more complex. Obviously, the results based on the SVM and LR algorithms are not good. Only the linear algorithm is then used for AAS pressure prediction and nonlinear properties are not further investigated.

To solve the issue above, we proposed a new approach to recover the in vivo perception function. In the designed system, self-packaging strain gauge sensors were used since they are small enough, low in cost, easily integrated into an in vivo device and, furthermore, are highly sensitive to pressure and insensitive to the ambient. In addition, long short-term memory models have been increasingly used in recent years to forecast in many fields, such as oil production [20,21], meteorological prediction [22,23], age estimation [24] and disease prediction [25,26]. Therefore, a long short-term memory algorithm was adopted since it shows excellent performance in processing nonlinear related features and very little degradation when coping with disturbance. Using the novel sensor system based on long short-term memory networks, the artificial anal sphincter could reconstruct an accurate in vivo perception system, and help patients towards a better life.

The rest of the article is organized as follows: Section 2 is an overview of strain gauge sensors and the AAS system with perception. Section 3 is devoted to the proposed method. Section 4 describes the data and analysis. Section 5 presents the experiment and results discussion. Finally, the work is concluded in Section 6.

## 2. Overview of Strain Gauge Sensors and AAS System with Perception System

**Figure 1:**
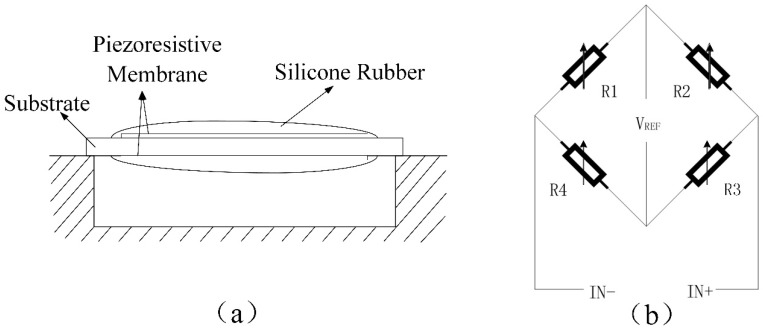
Self-packaging pressure sensor (**a**) and Wheatstone bridge (**b**).

A self-packaging strain gauge sensor system is designed, as shown in Figure 1a. Two strain gauge sensors and two matching resistors are typically connected to a Wheatstone Bridge electrically (see Figure 1b), which could increase the sensor sensitivity and compensates for temperature effects [27]. The self-packaging strain gauge sensor model is made of two piezoresistive membrane layers immersed in a substrate. It can measure the change in resistance using pressure. The designed sensor system is encased in a medical-grade silicone-filled membrane. Then, the sensor can be isolated from water, a major source of drift.

A Wheatstone bridge configuration could be used to calculate the sensitivity of the sensor from the gauge factor value. From Figure 1b, we can obtain the output voltage of the bridge $\;\Delta U_{O}$, sensitivity of voltage $S_{U}\;$ and nonlinear error of Wheatstone Bridge γ γ as,

(1) $$\left|{\mathrm{IN}}^{+}-{\mathrm{IN}}^{-}\right|=\Delta U_{O}=\frac{1}{2}\frac{\Delta R}{R}\cdot V_{REF}$$

(2) $$S_{U}=\frac{\Delta U_{O}}{\frac{\Delta R}{R}}=\frac{1}{2}\cdot V_{REF}$$

(3) $$\gamma =\frac{1}{2}\;\left(\frac{\Delta R_{1}}{R_{1}}+\frac{\Delta R_{2}}{R_{2}}+\frac{\Delta R_{3}}{R_{3}}+\frac{\Delta R_{4}}{R_{4}}\right)\times 100\%=0$$

where $V_{REF}$ is excitation voltage.

When forces are detected by the sensors, the strain gauges on both sides of substrate undergo opposite deformations, namely tensile strain and compression strain, respectively. Then, the effects of nonlinear errors can be eliminated. The strain gauge sensor has been validated for good sensing performance [28].

**Figure 2:**
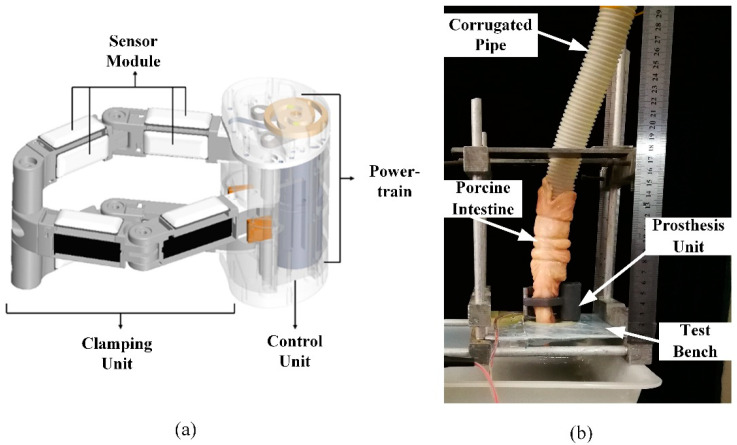
Schematic diagram of AAS system with sensor system (**a**) and work principle (**b**).

The artificial anal sphincter system has become the most popular method to treat fecal incontinence. The self-packaging strain gauge sensors are mounted into an Artificial Anal Sphincter (AAS) with a closed three-ring clamping mechanism to realize perception reconstruction when controlling defecation, which is shown in Figure 2a. The experimental platform for the working principle is presented in Figure 2b. Water is injected into the porcine intestine to simulate stool in it. The AAS system is used to control the contents in intestine by clamping and relaxing actions. In this process, the perception is reconstructed by pressure detection based on the proposed self-packaging sensor system. That is to say, patients with the AAS system could feel whether the rectum is full (feeling a high pressure) and defecate as desired.

## 3. Methodology

It has been demonstrated that there is a strong correlation between stool volume and intestinal pressure [19]. In research [19], a certain performance of perception reconstruction prediction is achieved, in which method pressure and the amount of feces are regarded as possessing a linear relationship. However, due to the complex conditions around the intestine, an accurate nonlinear relationship between them should be determined to improve the prediction of perception reconstruction. In addition, another obvious feature of the perception model is that the intestinal pressure of the current moment has a strong correlation with that of the previous moment. That is, the state of the current moment is not only affected by the current information, but is also affected by the previous state. Therefore, this should be considered in the perception model.

Because of a temporal correlation between the amount of feces at different times, the long short-term memory (LSTM) network, which shows an excellent performance in processing time series-related features and can better fit the time series features, is used in this article to improve the prediction accuracy of perception model.

### 3.1. Basic Theory of LSTM Neural Network

**Figure 3:**
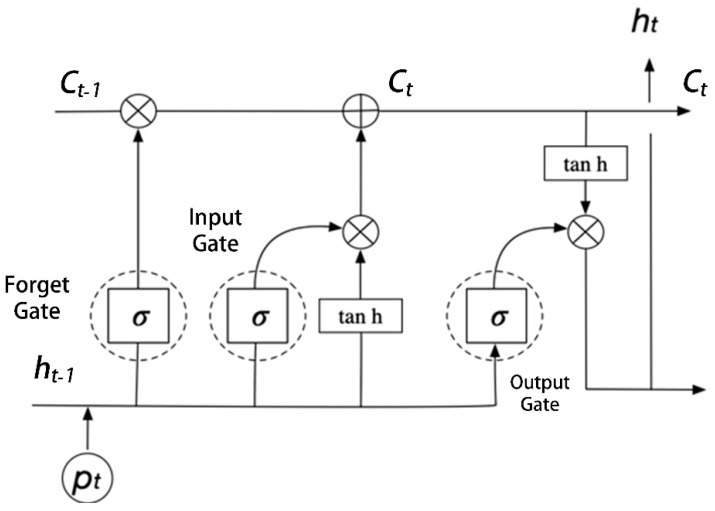
Internal structure of LSTM model.

The key to the accuracy of AAS perception reconstruction prediction lies in the LSTM model, whose internal structure is shown in Figure 3.

LSTM adopts the method of gated output, that is, three gates (forget gate, input gate, output gate) and two states (Cell State, Hidden State).

The forget gate layer is a sigmoid layer, which decides what information will be discarded from the cell state. The equation is as [29],

(4) $$f_{t}=\sigma \left(W_{f}\left[h_{t-1},p_{t}\right]+b_{f}\right)$$

where $f_{t}$ is forget gate output; $W_{f}$ is weight matrix; $h_{t-1}$ is hidden state of previous step; $p_{t}$ is input at current step; $b_{f}$ is bias.

The next step is to decide what new information will be stored in the cell state. First, a sigmoid layer called the “input gate layer” decides which values will be updated. Next, a tanh layer creates a vector of new candidate values, $\tilde{C_{t}}$, that could be added to the state. Both can be expressed as,

(5) $$i_{t}=\sigma \left(W_{i}\left[h_{t-1},p_{t}\right]+b_{i}\right)$$

(6) $$\tilde{C_{t}}=\tanh\left(W_{c}\left[h_{t-1},p_{t}\right]+b_{c}\right)$$

where $i_{t}$ represents probability of retaining information; $W_{i}$ and $W_{c}$ are weight matrices; $b_{i}$ and $b_{c}$ are biases. The two activation functions are utilized to enhance the nonlinearity of the network, and are expressed as:

(7) $$\sigma \left(x\right)=\frac{1}{1+e^{-x}}$$

(8) $$tanh\left(x\right)=\frac{e^{x}-e^{-x}}{e^{x}+e^{-x}}$$

In the next step, $i_{t}$ and $\tilde{C_{t}}$ will be combined to create an update to the state. The old cell state $C_{t-1}$ is multiplied $f_{t}$, forgetting the things we decided to forget earlier. Then $i_{t}\times \tilde{C_{t}}$ is added. These are the new candidate values, scaled by how much we decided to update each state value. We can obtain that the equation,

(9) $$C_{t}=f_{t}\times C_{t-1}+i_{t}\times \tilde{C_{t}}$$

Finally, the output will be based on cell state, but will be a filtered version. The output information $o_{t}$ is as follows,

(10) $$o_{t}=\sigma \left(W_{o}\left[h_{t-1},p_{t}\right]+b_{o}\right)$$

Then, the hidden state of current step $h_{t}$ can be deduced as,

(11) $$h_{t}=o_{t}\times \tanh\left(C_{t}\right)$$

where $W_{o}$ is the weight matrix and $b_{o}$ is bias.

The prediction output of current moment is shown in Equation (12),

(12) $${\hat{y}}_{t}=\sigma \left(Vh_{t}+c\right)$$

where 𝑉 is the parameter matrix.

Based on the above forward propagation formula, all parameters in the LSTM can be iterated through the gradient descent algorithm. The key to the gradient descent algorithm is to solve the gradients of $h_{t}$ and $C_{t}$. The derivation process of the gradients of $h_{t}$ and $C_{t}$ is as follows.

Define the loss function $L\left(t\right)$, as shown in Equation (13), where the loss at time 𝑡 is 𝑙(𝑡), and the loss in next step is $L\left(t+1\right)$.

(13) $$L\left(t\right)=\left\{\begin{array}{c} l\left(t\right)+L\left(t+1\right)\;\;if\;t<\tau \\ l\left(t\right)\;\;\;\;\;\;\;\;\;\;\;\;\;\;\;\;\;\;\;\;\;\;\;if\;t=\tau \end{array}\right.$$

The gradients of $h_{t}$ and $C_{t}$ are defined as,

(14) $$\delta_{h}^{\left(t\right)}=\frac{\partial L}{\partial h_{t}}$$

(15) $$\delta_{c}^{\left(t\right)}=\frac{\partial L}{\partial C_{t}}$$

The gradients of $h_{t}$ and $C_{t}$ at time τ can be expressed as,

(16) $$\delta_{h}^{\left(\tau \right)}={\left(\frac{\partial o_{\left(\tau \right)}}{\partial h_{\left(\tau \right)}}\right)}^{T}\frac{\partial L_{\left(\tau \right)}}{\partial o_{\left(\tau \right)}}=V^{T}\left({\hat{y}}_{\left(\tau \right)}-y_{\left(\tau \right)}\right)$$

(17) $$\delta_{c}^{\left(\tau \right)}={\left(\frac{\partial h_{\left(\tau \right)}}{\partial C_{\left(\tau \right)}}\right)}^{T}\frac{\partial L_{\left(\tau \right)}}{\partial h_{\left(\tau \right)}}=\delta_{h}^{\left(\tau \right)}*o_{\left(\tau \right)}*\left(1-\tanh^{2}\left(C_{\left(\tau \right)}\right)\right)$$

Furthermore, use $\partial h_{\left(t+1\right)}$ to inversely derive $\partial h_{\left(t\right)}$, as shown,

(18) $$\delta_{h}^{\left(t\right)}=\frac{\partial l_{\left(t\right)}}{\partial h_{\left(t\right)}}+{\left(\frac{\partial h_{\left(t+1\right)}}{\partial h_{\left(t\right)}}\right)}^{T}\frac{\partial L_{\left(t+1\right)}}{\partial h_{\left(t+1\right)}}=V^{T}\left({\hat{y}}_{\left(t\right)}-y_{\left(t\right)}\right)+{\left(\frac{\partial h_{\left(t+1\right)}}{\partial h_{\left(t\right)}}\right)}^{T}\delta_{h}^{\left(t+1\right)}$$

where, $\frac{\partial h_{\left(t+1\right)}}{\partial h_{\left(t\right)}}$ can be derived from Equation (19),

(19) $$\begin{array}{cc} \frac{\partial h_{\left(t+1\right)}}{\partial h_{\left(t\right)}}= & diag\left[o_{\left(t+1\right)}*\left(1-o_{\left(t+1\right)}\right)*\tanh\left(C_{\left(t+1\right)}\right)\right]W_{o}\\ & \;\;\;\;\;\;\;\;\;+diag\left[\Delta C*f_{\left(t+1\right)}*\left(1-f_{\left(t+1\right)}\right)*C_{\left(t\right)}\right]W_{f}\\ & \;\;+diag\left\{\Delta C*i_{\left(t+1\right)}*\left[1-{\tilde{C}}_{\left(t+1\right)}^{2}\right]\right\}W_{a}\\ & \;\;\;\;\;\;\;\;\;\;\;\;+diag\left[\Delta C*{\tilde{C}}_{\left(t+1\right)}*i_{\left(t+1\right)}*\left(1-i_{\left(t+1\right)}\right)\right]W_{i} \end{array}$$

Similarly, $\delta_{C}^{\left(t+1\right)}$ can be obtained from $\delta_{C}^{\left(t\right)}$,

(20) $$\begin{array}{cc} \delta_{C}^{\left(t\right)}= & {\left(\frac{\partial C_{\left(t+1\right)}}{\partial C_{\left(t\right)}}\right)}^{T}\frac{\partial L}{\partial C_{\left(t+1\right)}}+{\left(\frac{\partial h_{\left(t\right)}}{\partial C_{\left(t\right)}}\right)}^{T}\frac{\partial L}{\partial h_{\left(t\right)}}\\ & ={\left(\frac{\partial C_{\left(t+1\right)}}{\partial C_{\left(t\right)}}\right)}^{T}\delta_{C}^{\left(t+1\right)}+\delta_{h}^{\left(t\right)}*o_{\left(t\right)}*\left(1-\tanh^{2}\left(C_{\left(t\right)}\right)\right)\\ =\delta_{C}^{\left(t+1\right)}*f_{\left(t+1\right)} & +\delta_{h}^{\left(t\right)}*o_{\left(t\right)}*\left(1-\tanh^{2}\left(C_{\left(t\right)}\right)\right) \end{array}$$

### 3.2. The Perception Model Based on LSTM Network

**Figure 4:**
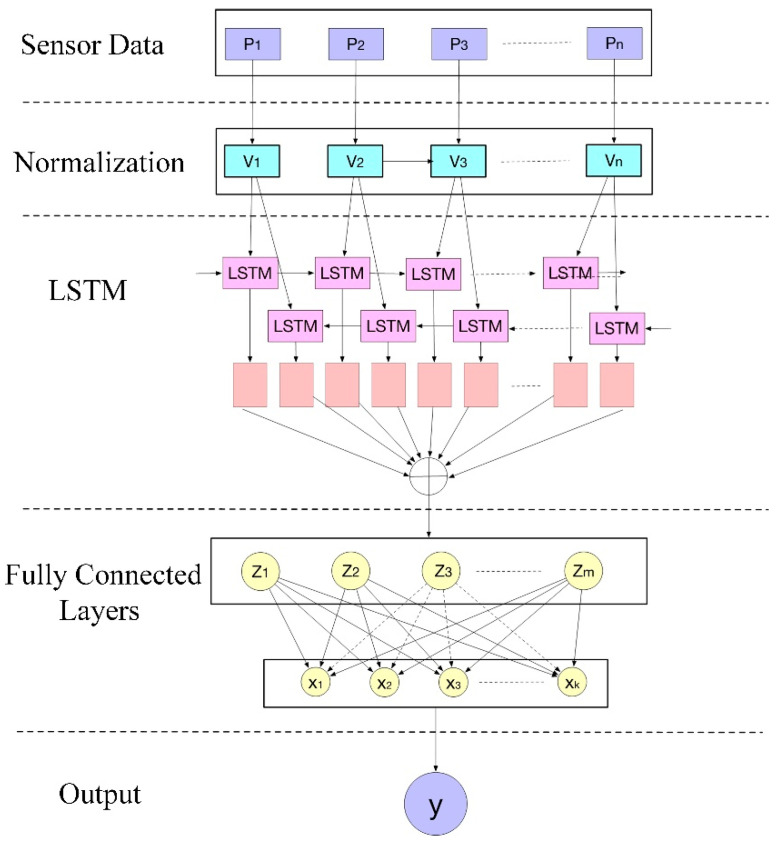
Defecation perception solution based on LSTM.

As is known, long short-term memory networks can effectively prevent the underfitting problem. In addition, using the dropout algorithm [30], the overfitting problem can be prevented, with a probability of dropout of 30%. In this study, the LSTM algorithm is used to assist the AAS system in the perception reconstruction. The overall scheme of the sensor system for perception reconstruction based on LSTM Networks is shown in Figure 4.

The sensor data will be normalized before feeding into the LSTM. A set of gates can maintain information in memory for long periods of time. The LSTM network enables faster and better training on long sequence data and avoids the problem of vanishing gradients. As a result, the model has speedier convergence. The fully connected layers map the output of the LSTM layer to the desired output size. In fully connected layers, all the inputs from one layer are connected to every activation unit of the next layer. For the last layer, the output is the prediction of feces amount (volume).

The perception model explores the mapping relationship between the data collected by the sensors and the amount of feces in intestine. Using the method above, the sensor perception model can be obtained by offline learning based on in vitro datasets. Once the model has been trained, you can use it to reason over data that it has not seen before, and to make predictions about those data. Here, the perception model can be used to predict the amount of stool based on the pressure data. Then, a defecation reminder will be issued on demand after comparing the detected pressure data with the preset defecation threshold.

### 3.3. System (Model) Evaluation Criteria

The Root-Mean-Square Error, as a quantitative evaluation index, is adopted to evaluate the accuracy of the model. The equation is presented as,

(21) $$\mathrm{RMSE}=\frac{1}{n}\sqrt{\frac{\sum {\left(m-\hat{m}\right)}^{2}}{n}}$$

where 𝑚 is the actual amount of feces in intestine; *$\hat{m}$* is predicted amount of feces; 𝑛 is the number of tested samples. A low RMSE value indicates that the simulated and observed data are close to each other, showing better accuracy. Thus, the lower the RMSE, the better the model performance.

## 4. Data and Analysis

### 4.1. Data Collection

The data source obtained by the AAS system proposed in this paper is the pressure data detected by eight self-packaging strain gauge pressure sensors, which are located at different positions on the clamping unit. Pressure data from each sensor can be regarded as relatively independent pressure vectors. The eight-channel strain gauge sensor layouts are listed in Table 1.

### 4.2. Normalization

The eight channels of pressure data collected by perception model have different value ranges. In order to speed up the model training and help gradient descent to converge faster, normalization is needed for the sensor data of each channel. The normalized value s can be deduced as follows,

(22) $$s=\frac{s_{i,j}-s_{i,min}}{s_{i,max}-s_{i,min}}$$

where $s_{i,j}$ is the 𝑗th sample data of the 𝑖th sensor; $s_{i,max}$ and $s_{i,min}$ are the maximum and minimum values of the 𝑖th sensor, respectively.

## 5. Case Studies (Results and Discussion)

In this section, experiments on the training and prediction process are conducted.

In the training process, data obtained from in vitro experiments are taken as the training set, while the testing set is composed of in vitro and in vivo experimental data. The sensor data is used as the input of training, and the mass of intestinal content is used as the label.

In prediction process, using the sensor data of in vitro and in vivo experiments as input, the mass of feces in the intestine is predicted to verify the feasibility of proposed perception model based on self-packaging strain gauge sensors. In order to prove the advantages of the LSTM model in terms of perception, the proposed model is also compared with the previous one based on linear regression.

### 5.1. Experiments Details

#### 5.1.1. In Vitro Experiment

The experimental platform was built, as shown in Figure 1b. The intestine was clamped by AAS system. Then, water was injected into the intestine from 0 g to 300 g. The water mass and the corresponding sensor readings were then recorded. These steps were repeated ten times.

The sample data are normalized by Equation (22) squashing to the range (0,1). The actions “open” and “close” of the AAS system are added to the training process as features, designated ‘1’ and ‘0’, respectively. Some of the data after preprocessing are shown in Table 2. The change in the amount of feces can be regarded as a time series. The amount of feces at the current moment is related to it at the previous moment. Therefore, the amount of feces at previous moment is also included as a feature. In the Table 2, P1–P8 represent the sensor data; $AAS_{S}$ represents the state of clamping unit; $M_{t}$ and $M_{t-1}$ represent the amount of feces at the current and previous moments, respectively.

#### 5.1.2. In Vivo Experiment

**Figure 5:**
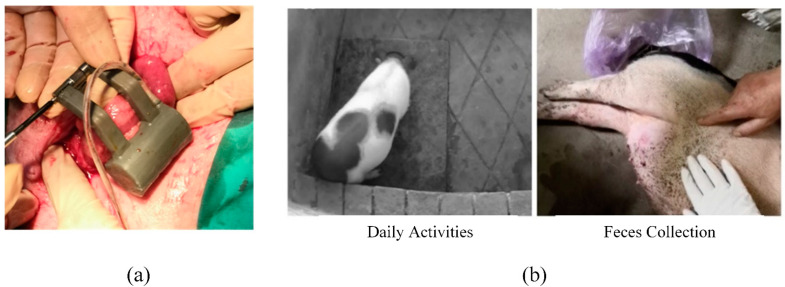
Implantation surgery of AAS with proposed sensor system (**a**) and in vivo experiments observation (**b**).

To further quantify the predictive performance of the models, Bama piglets were used for the in vivo experiments. The AAS system with the proposed perception reconstruction model was implanted in the piglet. Figure 5a shows the implantation surgery. The anal sphincter was replaced by the AAS system.

In this test, piglets were fed 600 g per day. The threshold value in the controller was set to 150 g. That is to say, when the feces mass detected by the perception model reaches a threshold value of 150 g, a warning for defecation is reminded on the controller. As a warning occurs, the AAS begins to open. Feces are collected for weighing and recording, which is used to verify the prediction accuracy of the defecation perception model. The observation of in vivo experiments is shown in Figure 5b.

### 5.2. Evaluation of Perception Reconstruction

In order to prove the advantages of the LSTM model in terms of perception, the proposed model is also compared with the previous one based on linear regression.

#### 5.2.1. Perception Reconstruction Based on Linear Regression Model

Linear regression (LR) model can be expressed by equations as,

(23) $$\left\{\begin{array}{c} \begin{array}{c} m_{1}=w_{1}p_{1}^{1}+w_{2}p_{2}^{1}+w_{3}p_{3}^{1}+w_{4}p_{4}^{1}+w_{5}p_{5}^{1}+w_{6}p_{6}^{1}+w_{7}p_{7}^{1}+w_{8}p_{8}^{1}\\ m_{2}=w_{1}p_{1}^{2}+w_{2}p_{2}^{2}+w_{3}p_{3}^{2}+w_{4}p_{4}^{2}+w_{5}p_{5}^{2}+w_{6}p_{6}^{2}+w_{7}p_{7}^{2}+w_{8}p_{8}^{2} \end{array}\\ \begin{array}{c} \vdots \\ m_{n}=w_{1}p_{1}^{n}+w_{2}p_{2}^{n}+w_{3}p_{3}^{n}+w_{4}p_{4}^{n}+w_{5}p_{5}^{n}+w_{6}p_{6}^{n}+w_{7}p_{7}^{n}+w_{8}p_{8}^{n} \end{array} \end{array}\right.$$

where $P_{i}^{j}$ is the 𝑗th sample datum of the 𝑖th sensor; $m_{j}$ is the 𝑗th reading of content mass in intestine; $w_{i}$ is a parameter.

**Figure 6:**
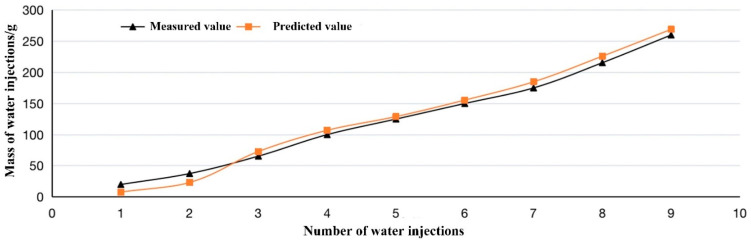
Prediction performance of LR model.

Through the gradient descent algorithm, the parameters of the linear regression model corresponding to Equation (23) can be obtained by training with the in vitro experimental data. The parameters indicate the relationship between the pressure matrix and the mass of feces. The pressure data were used as the input, then the mass of feces was predicted. Figure 6 presents the difference between the predicted and the measured value. When the mass of intestinal content is more than 50 g, the body begins to sense a need to pass stool. Considering these circumstances, it can be seen from Figure 6 that the relative error between the predicted and the measured value is 10%, and the RMSE value is 9.379.

#### 5.2.2. Perception Reconstruction Based on LSTM

**Figure 7:**
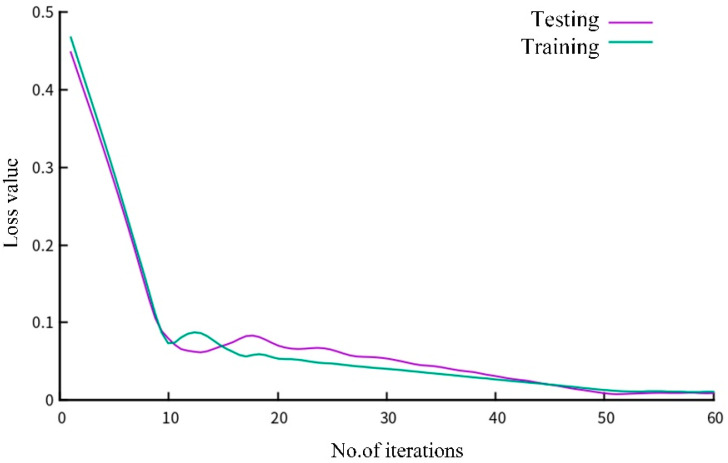
Loss comparison between testing and training dataset of LSTM model.

In order to train LSTM networks, the Keras toolbox is used with a mean-absolute error function and an Adam algorithm. The loss values of the testing dataset and the training dataset are shown in Figure 7. The results demonstrate that the proposed perception model has a good convergence after 60 iterations.

**Figure 8:**
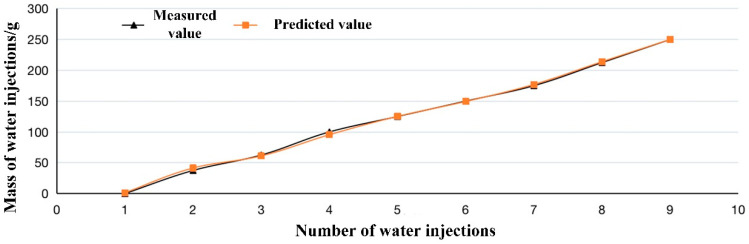
Prediction performance of LSTM model.

The trained LSTM perception model is used to predict the actual value (i.e., mass of injected water), based on the sensor pressure data collected in the in vitro experiments. Figure 8 shows the forecasting curve and the actual curve of injected water. The RMSE value is 2.906. With the comparison of LR and LSTM for prediction of injected water mass, LSTM based approach performs better than LR system, by increasing the accuracy rate of 69% in terms of RMSE on predicted water mass using test in vitro data.

**Figure 9:**
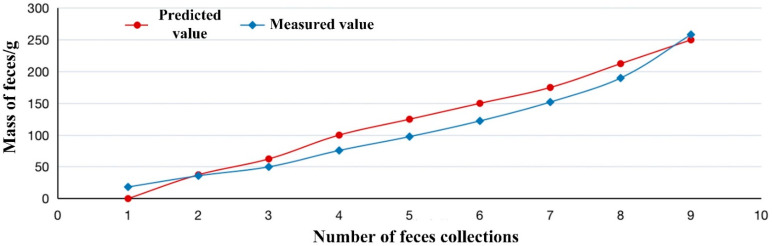
Defecation prediction in vivo experiment based on proposed LSTM model.

For further evaluation, the proposed perception model based on LSTM has been used for prediction in vivo experiments. Results are shown in Figure 9, in which the prediction curve of our proposed model has the same trend as the actual curve. There is only slightly worse performance than that in vitro experiment. It should be noted that the prediction values are larger than the actual ones. This is because of different physiological mechanisms of intestine between in vitro and in vivo experiments. In in vivo experiments, the sensor system usually detects higher pressure than intestinal contents provide due to the animal’s own daily activities, such as respiration and walking.

Although the dataset for training the LSTM-based perception model in this paper does not include data from in vivo experiments, the proposed perception model can still accurately predict the mass of intestinal feces, which shows that the LSTM model has good robustness.

## 6. Conclusions

To the best of our knowledge, the measurement of distributed and integrated strain gauge sensors in artificial anal sphincters is yet to be realized. This work investigates a long short-term memory network-based sensor system using self-packaging strain gauge sensors for in vivo perception reconstruction. The sensor features that yield the highest sensitivity are demonstrated and discussed in terms of the mechanical and electrical properties. In addition, the self-packaging sensor system was designed efficiently to be deployed in an artificial anal sphincter system to detect pressure in the intestine. In particular, a traditional linear regression approach was also applied to the sensor system as a comparative forecasting model.

In this study, the proposed system has been tested. Compared with the traditional linear regression approach using data from in vitro experiments, the results using LSTM based system show that the Root-Mean-Square Error is reduced by more than 69%. It demonstrates that the prediction accuracy of the proposed LSTM model is higher than that of the LR model to reach a more accurate prediction of the amount of intestinal content. For further evaluation, the proposed perception reconstruction model was tested in vivo for more than 60 days in porcine models simulating fecal incontinence. The proposed system can estimate the amount of the contents in intestine and shows very little degradation when coping with disturbance, such as respiration and walking. All of the above indicate a good performance on prediction and robustness of the designed sensor system through the experiments with limited data. Furthermore, a good reference can be provided by the findings for future selection of prediction models and establishments of warning systems for in vivo devices.

## Figures and Tables

**Table 1:** Eight-channel strain gauge sensor layout.

Name	No.	Location
Sensor 1	p1	Upper arm, radial 1
Sensor 2	p2	Upper arm, axial 1
Sensor 3	p3	Upper arm, radial 2
Sensor 4	p4	Upper arm, axial 2
Sensor 5	p5	Middle arm, radial 1
Sensor 6	p6	Middle arm, axial 1
Sensor 7	p7	Middle arm, radial 2
Sensor 8	p8	Middle arm, axial 2

**Table 2:** Some of the data after convenience perception model preprocessing.

$M_{t-1}$	P1	P2	P3	P4	P5	P6	P7	P8	$AAS_{S}$	$M_{t}$
1.000	0.059	0.025	0.115	0.013	0.067	0.084	0.135	0.110	1.0	0.000
0.000	0.053	0.006	0.093	0.013	0.118	0.148	0.187	0.137	0.0	0.150
0.150	0.099	0.054	0.117	0.073	0.205	0.228	0.257	0.195	0.0	0.250
0.250	0.226	0.184	0.197	0.203	0.353	0.355	0.375	0.316	0.0	0.400
……	……	……	……	……	……	……	……	……	……	……
0.700	0.778	0.738	0.724	0.713	0.823	0.818	0.815	0.803	0.0	0.850
0.850	0.999	0.937	0.999	0.890	0.999	1.000	0.996	0.999	0.0	1.000

## Data Availability

Not applicable.
